# Ligating the varicose vein trunk for bleeding stomal varices in portal hypertension: a case report

**DOI:** 10.3389/fmed.2024.1483261

**Published:** 2025-01-07

**Authors:** Jialiang Sun, Zhanhai Tan, Jifa Zhang

**Affiliations:** Department of General Surgery, Shanghai Fengxian District Central Hospital, Shanghai, China

**Keywords:** bleeding stoma varices, colostomy, portal hypertension, varicose vein trunk, decompensated cirrhosis

## Abstract

**Introduction:**

In colostomy-related complications, variceal hemorrhage particularly induced by cirrhosis and portal hypertension is seldom encountered. The onset of peristome variceal hemorrhage necessitates swift and effective intervention to prevent potentially life-threatening outcomes such as hemorrhagic shock and recurrent stoma bleeding.

**Case presentation:**

This report details a case of repeated varicose vein hemorrhage around the stoma in a patient with liver cirrhosis. Abdominal enhanced CT images revealed that the stomal varices originated from a branch of the inferior mesenteric vein, with vein balls encompassing the stoma. The patient was acquired with successful hemostasis through high ligation of the various vein primary trunk, and stripping of the vein balls around stoma, along with intracutaneously suturing of the sub-abdominal wall varicose veins. When the stoma bag was changed it was observed that the skin surrounding the stoma was flat, the mucosa was red, and the varicose venous mass had vanished. After 2 months of follow-up, the stomal function was doing well without any rebleeding episodes.

**Conclusion:**

In this instance, decompensated cirrhosis led to stomal varices and recurrent bleeding, which was initially managed with local compression and suture therapy but resulted in rebleeding. Our team’s approach through blocking the primary trunk of variceal vein via a minimal incision under local anesthesia may offer a new treatment strategy for patients with poor liver function who cannot withstand the trauma associated with general anesthesia and major surgery.

## 1 Introduction

In colostomy-related complications, variceal hemorrhage particularly induced by cirrhosis and portal hypertension is seldom encountered. This type of bleeding typically occurs at the skin and mucous membrane junction, and often result in substantial blood loss compared to ordinary colostomy bleeding which usually originates within the colostomy mucosa and involves less blood (1). Clinicians must accurately discern the nature of hemorrhage in colostomy patient. Conventional colostomy mucosal bleeding can be controlled through the application of pressure, topical hemostatic agents, suturing, or electrocoagulation. However, varicose vein bleeding tends to be refractory problem and uncontrollable rebleeding episodes through these measures (2). In severe instances, patients might face the risk of hemorrhagic shock, which significantly diminish their quality of life. Through comprehensive literature review, there have been few approaches to treat local bleeding and portal hypertension.

We present a case involving a patient with liver cirrhosis and portal hypertension who experienced rebleeding episodes in varicose vein around the stoma. Successful hemostasis was ultimately achieved by ligating the varicose vein trunk, removing the varicose vein balls, and simultaneously performing intracutaneous suturing of the colostomy abdominal wall varices. It is worth popularizing for patients with poor liver function offering a new treatment strategy.

### 1.1 Clinical data

A 70-year-old male patient was admitted to the emergency room with a 4-h history of colostomy varices bleeding. The patient had two-year history of cirrhotic ascites and portal hypertension, and long term of cirrhosis after hepatitis B, hypertension and type 2 diabetes mellitus. He underwent radical resection of a rectal tumor and colostomy in 2013. Over the past year, he had experienced rebleeding episodes in stoma and received multiple local suture treatments in the emergency room of our hospital.

**Figure 1:**
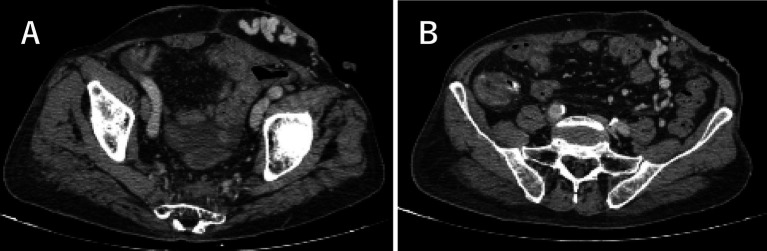
The transverse sections of whole abdominal enhanced CT images. **(A)** Dilated and tortuous vein shadows visible around the stoma (blue arrow). **(B)** The main trunk of the varicose vein around the stoma was located in the left branch of the inferior mesenteric vein (red arrow).

Whole Abdominal Enhanced CT revealed dilated and tortuous vein shadows around the stoma on left abdominal wall visibly. The inferior mesenteric vein communicates with the inferior epigastric vein around the stoma, and the main trunk of the varicose vein was located in the left branch of the inferior mesenteric vein (Figure 1). Biochemical tests showed: Alanine Aminotransferase (ALT): 28 U/L; Aspartate Transaminase (AST): 33 U/L; Total bilirubin (T-Bil): 10.8 umol/L; Albumin: 27.4 g/L. Blood routine examination results were as follows: White blood cell (WBC) count: 9.3*10^9/L; Red blood cell (RBC) count: 2.3*10^12/L; Hemoglobin (Hb): 72 g/L; Platelet (PLT) count: 153*10^9/L. Coagulation function tests indicated Thrombin time (TT) of 12.5 s.

**Diagnosis**: stoma hemorrhage; cirrhotic ascites; portal hypertension; cirrhosis after hepatitis B; anemia; hypoproteinemia; hypertension; type 2 diabetes mellitus. The Child-Pugh score was assessed as grade B with a score of 9.

#### 1.1.1 First operation after admission

**Figure 2:**
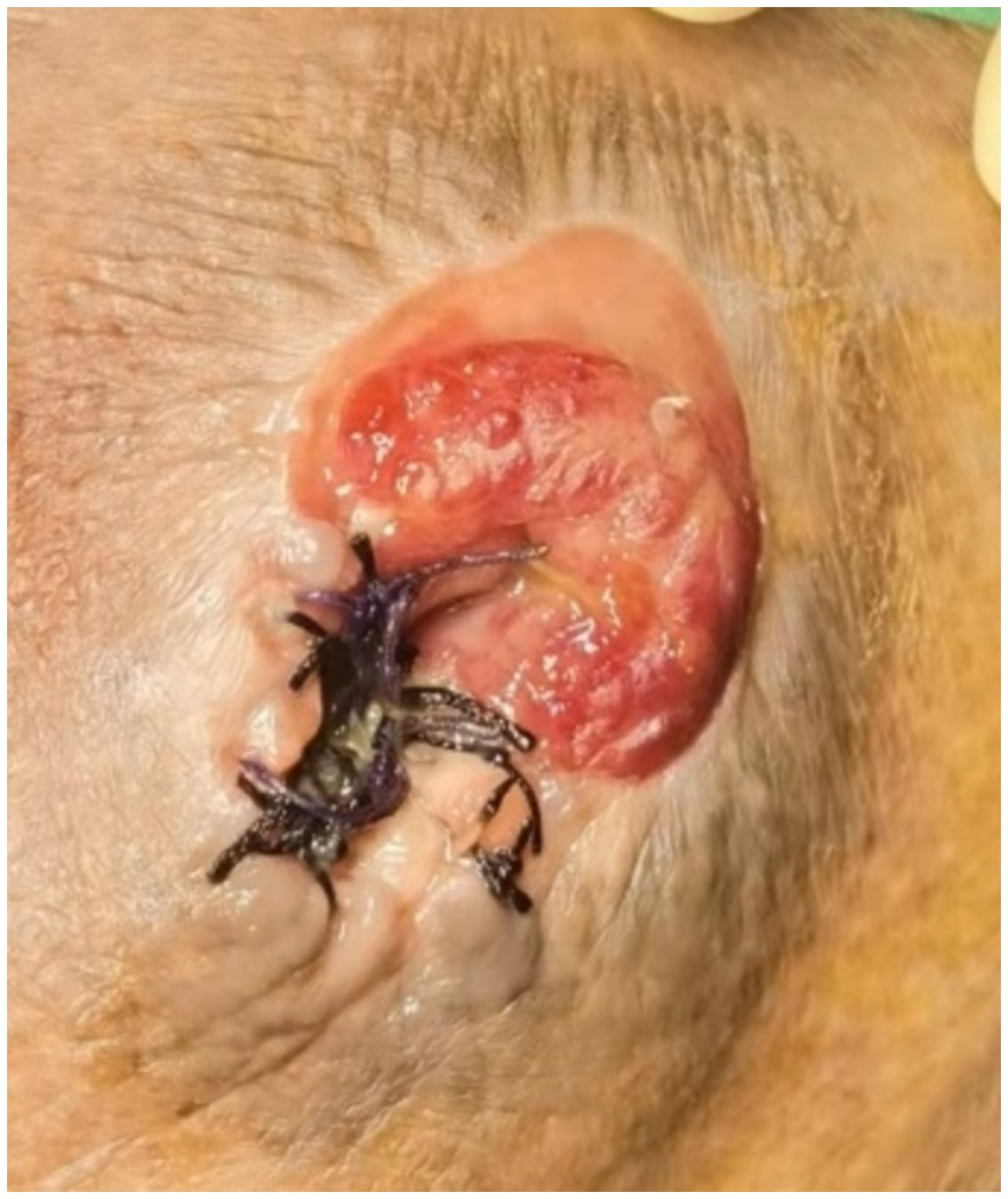
The tightened suturing and ligating in stoma bleeding point.

Stomal varicose vein bleeding was observed in the local mucocutaneous junction on 27-October-2021. Therefore, emergency hemostasis was achieved through tightly suturing the bleeding point in the stoma. Approximately two-thirds of the stoma was retained, which residual diameter was about 2 cm (Figure 2).

#### 1.1.2 Second operation after admission

**Figure 3:**
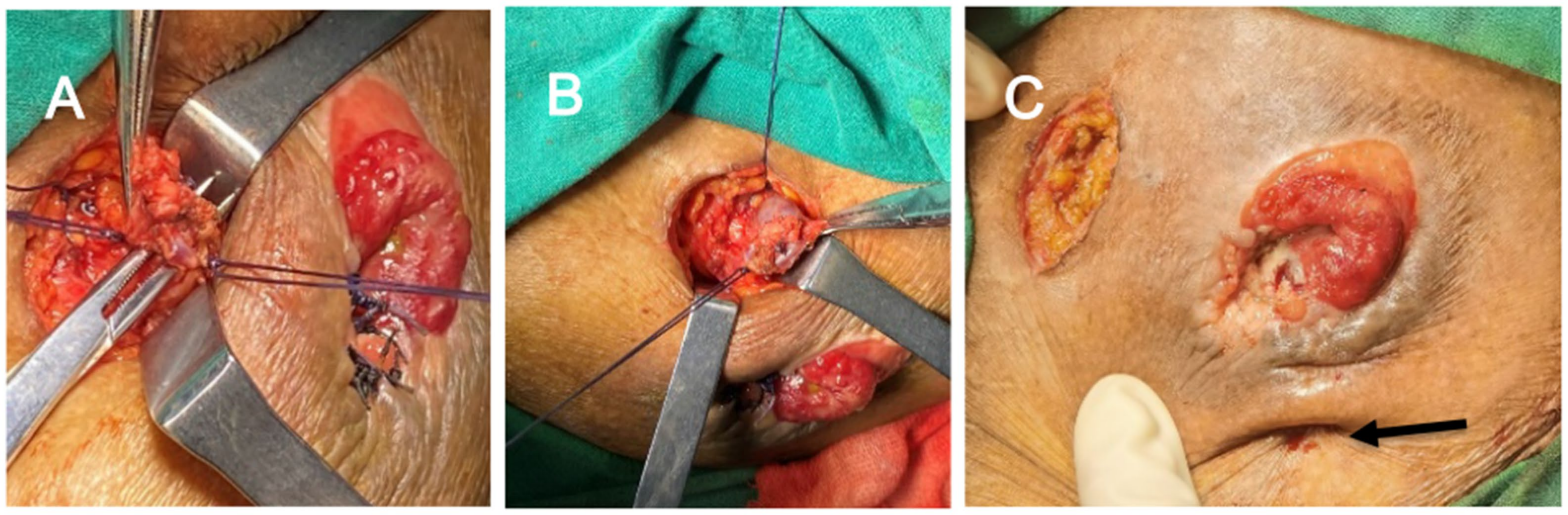
The findings of situation in surgery. **(A)** High ligation of the left branch of the inferior mesenteric vein. **(B)** The varicose bulb surrounding the stoma was stripped. **(C)** The subcutaneous soft tissues and veins were fixed by tying them *in situ* (black arrow).

After about 2 weeks, rebleeding from the stoma was located at the suture site of the previous operation. Combined with whole abdominal enhanced CT images, the main trunk of the varicose vein around the stoma was located in the left branch of the inferior mesenteric vein, which was dilated and communicated with the inferior epigastric vein. Based on this analysis, an oblique incision was made about 4 cm from 3 cm above the stoma under local anesthesia. After separating the subcutaneous tissue, more varicose veins were observed, which were proximally separated to the junction of the left branch of the inferior mesenteric vein and the peritoneum. Opening the peritoneum, clear peritoneal effusion was released about 400 mL. At this point, the left branch of the inferior mesenteric vein was transected and the varicose bulb surrounding the stoma was dissected. Considering that there were few varicose veins under the stoma, it was not conducive to seal and close the stoma bag after incision. Therefore, the varicose veins under the stoma were sutured intradermally with a proline 2–0, and the subcutaneous soft tissues and veins were fixed by tying them *in situ*. At the same time, removing the first suture around the stoma, there was no obviously bleeding in the mucosa, and the surrounding skin of stoma was wrinkled (Figure 3).

#### 1.1.3 Follow-up

**Figure 4:**
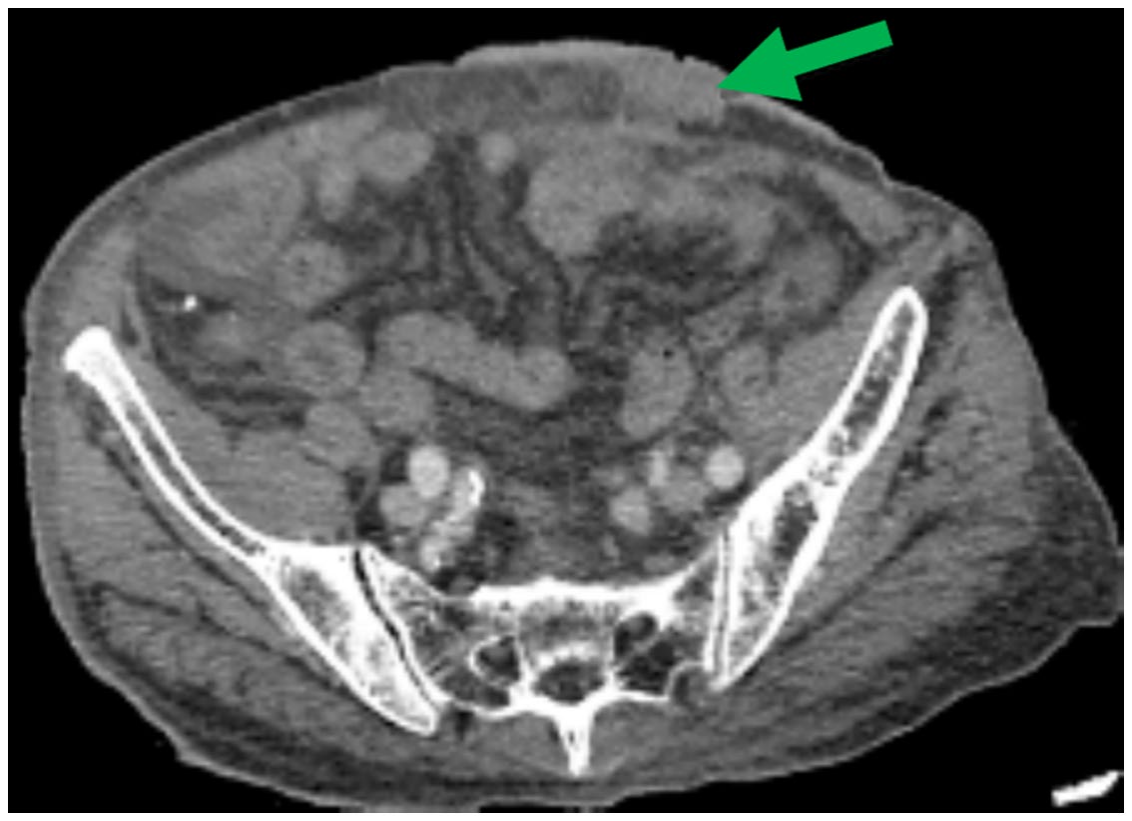
Postoperative enhanced abdominal CT revealed a significant disappearance of most varicose veins around the stoma (green arrow).

**Figure 5:**
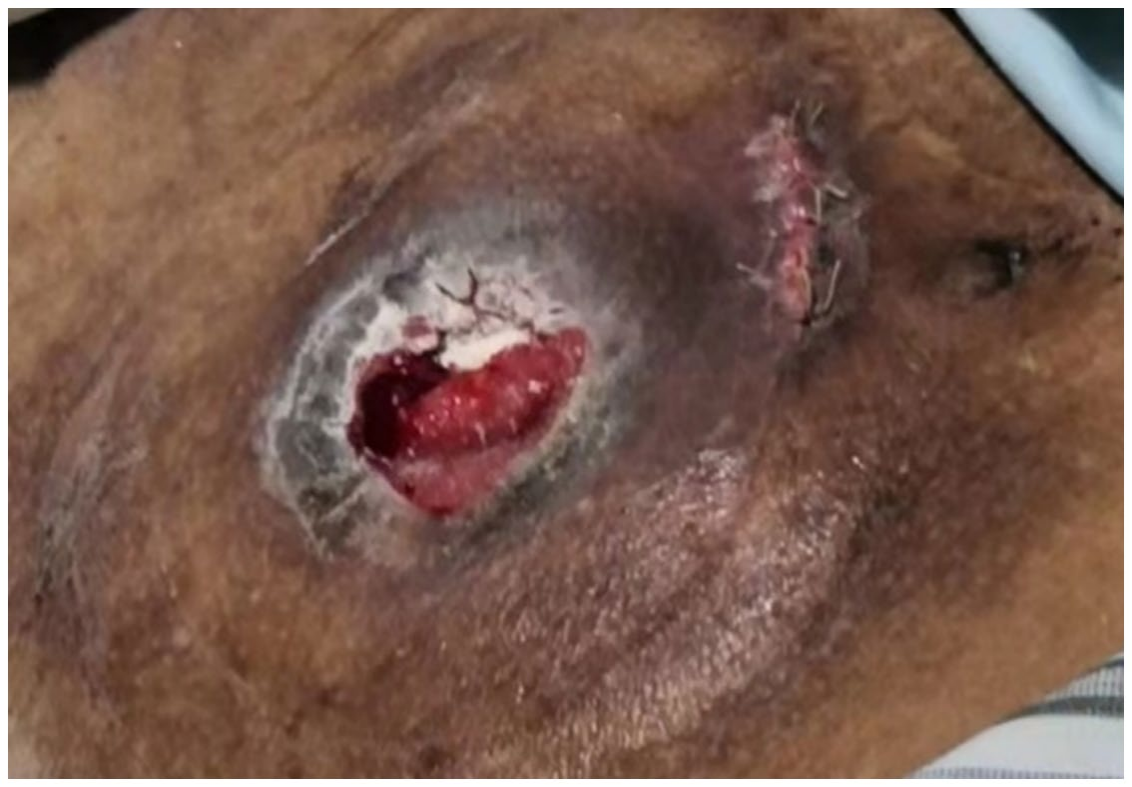
Six days post-surgery, the skin surrounding the stoma was flat, the mucosa was red, and the varicose venous mass had vanished.

Postoperative Enhanced Abdominal CT revealed a significant disappearance of most varicose veins around the stoma (Figure 4). The patient’s ascites and hypoproteinemia were improved after receiving blood transfusions, protein infusions, and both enteral and parenteral nutritional support during hospitalization. Six days post-surgery, when the stoma bag was changed it was observed that the skin surrounding the stoma was flat, the mucosa was red, and the varicose venous mass had vanished (Figure 5). After 2 months of follow-up, the stomal function was doing well without any rebleeding episodes but the patient’s condition and cirrhosis had markedly deteriorated due to anorexia, fatigue, malnutrition, fullness of abdomen, weight loss and renal failure. The patient ultimately decided to give up future treatment.

## 2 Discussion

Among the complications associated with colostomy, variceal bleeding is a rare occurrence, typically linked to cirrhosis and portal hypertension (1). Fucini et al. suggested that patients with liver cirrhosis should avoid colostomy and opt for anastomosis during colon surgery as much as possible (3). The pathogenesis of varicose vein masses involves two different pressure venous system including the high pressure of the portal vein system and the low pressure of the systemic circulation, which communicate with each other in the peristomal veins (4). According to literature reports, peristomal varicose vein bleeding with cirrhosis generally occurs 48 months post-operation, but can also happen as early as 5 months after surgery (5).

For bleeding from stoma varices, local treatments such as compression, suturing, electrocoagulation, and application of hemostatic agents are commonly performed. However, these methods have a higher rate of rebleeding (1). Some specialists advocate for reconstruction of the stoma, separation of the junction of skin and mucosa around the stoma, and local injection of sclerosing agents. However, these approaches do not fundamentally address the issue of varicose veins around the stoma caused by high pressure. For patients with Child-Pugh grade A liver function, portosystemic shunt or devascularization procedures can be performed to significantly reduce portal hypertension. This can decrease the probability of rebleeding from the stoma by 50%. For patients in poor general health, transjugular intrahepatic portosystemic shunt (TIPS) can be a viable option to reduce portal hypertension and control acute bleeding, which could achieve the rate of hemostasis by 66% and reduce the likelihood of rebleeding by 78.5% (6, 7). Other new interventional methods for treating varicose bleeding, including retrograde venous embolization with sclerosis or balloon closure and ultrasound-guided thrombin injection, may have partial efficacy (5, 8, 9). For patients with advanced liver cirrhosis and liver failure, the most fundamental treatment strategy is liver transplantation. However, there are no relevant reports on blocking the main trunk of variceal vein through a small incision under local anesthesia to mitigate variceal vein bleeding after an extensive literature review. Therefore, we describe it as follows.

In this case, the patient with cirrhosis after hepatitis B had progressed to portal hypertension along with cirrhotic ascites 2 years ago and had undergone Hartmann’s procedure for rectal cancer before 8 years. Over the past year, the patient experienced recurrent bleeding around the stoma, which was temporarily alleviated with compression or local suturing. However, as liver cirrhosis aggravated and generated ascites and hypoproteinemia, the patient’s liver function deteriorated and portal hypertension worsened (10). It resulted in more prominent varicose veins around the stoma and rebleeding so that simple suturing could not fundamentally resolved the problem. Given poor liver function, the patient was not a candidate for portosystemic shunting or devascularization surgery to reduce portal hypertension. In view of the potential risk that TIPS could exacerbated hepatic encephalopathy and thromboembolism, interventional therapy was not suitable for this patient.

Through preoperative analysis of enhanced abdominal CT images, it was determined that the main varicose veins surrounding the stoma originated from a branch of the inferior mesenteric vein. Based on these findings, the surgical team proceeded to ligate the branch of the inferior mesenteric vein and excised the varicose mass through a minimal incision under local anesthesia. Concurrently, the varicose vein beneath the stoma was sutured intermittently in the intradermal layer to block its communication with the inferior epigastric vein. Intraoperative observation of the skin folds around the stoma revealed no rebleeding following the removal of the previous sutures. Postoperative re-examination with enhanced abdominal CT imaging confirmed significant disappearance of the varicose veins around the stoma. Upon changing the stoma bag, it was noted that the skin surrounding the stoma became flat, and the mucosa appeared healthy and ruddy. After 2 months of follow-up, the stomal function was doing well without any rebleeding episodes.

In conclusion, TIPS may pose or exacerbate the risk of hepatic encephalopathy and thromboembolism in patients with decompensated cirrhosis. Those who have poor liver function may not tolerate the trauma associated with general anesthesia and major surgery. Our team has reported that the therapy can reduce the risk of peristomal variceal bleeding by ligating the main trunk of variceal vein using a small incision under local anesthesia. This may offer an alternative treatment strategy for such patients.

## Data Availability

The datasets presented in this study can be found in online repositories. The names of the repository/repositories and accession number(s) can be found in the article/supplementary material.
